# Alveolar Abscess

**Published:** 1879-06

**Authors:** M. F. Finley


### THE DENTAL REGISTER.

VOL. XXXIII.] JUNE, 1879. No 6.

### ALVEOLAR ABSCESS.

BY M. F. FINLEY, D. D. S.

*Read before the Michigan State Dental Association.*

*Mr. President and Gentlemen:*—I have chosen for a theme alveolar abscess. This has been, and no doubt will ever be, a source of great annoyance to the practitioner as well as to the patient; on account of the unwillingness of the patient to submit to a proper and systematic course of treatment, which oftentimes becomes odious unless he stops to consider the results of the disease if left to follow its course, as compared with the widely different issue of the case if properly and successfully managed: and also on account of the persistent course of treatment required in the majority of cases which present themselves; and the amount of suffering and inconvenience this disease causes the patient. It also is a great incentive to become proficient in the management of this disease, because of its difficulties and the bearing it has upon the comforts of humanity.

The proximate cause of alveolar abscess is destructive inflammation of the periosteum, or more explicitly, inflam mation of the investing membrane of the tooth of sufficient duration and severity to result in checking life action in the tissue. It then undergoes solution, and the nutrient material brought into the dieased territory is for the most part vitiated and is added to the accumulating debris.

This cause has its origin in others more remote, always occurring subsequent to, and in the vast majority of cases, resulting from the death of the pulp. Also from dead and degenerating teeth, mechanical violence, as blows on the teeth, accidental or otherwise; the too rapid forcing of the teeth apart; sudden and extreme thermal changes; the presence of fillings forced through the root of a tooth; and in a great many cases where undue pressure is exerted upon an exposed pulp. The course of the disease from its incipient stages, I will try to describe in a brief form.

The presence of the irritating cause produces at first a slight determination of blood to the part and an increased flow through the vessels, causing distension of the capillaries; reaction then takes place in these, resulting in stasis of blood, and exudation begins. But the irritating cause still exerting its influence, the increased flow of blood is kept up, producing a slight sense of fullness, which invites contact, and even pressure, from the opposing teeth, causing a rather pleasurable sensation and seeming relief.

As a consequence of the partial stasis and increased flow of blood, the surroundings precluding free expansion of the capillaries and other small vessels ramifying this membrane. these structures are pressed upon proportionally to the force of the determination of blood to the part.

This excessive pressure results in free exudation, and also in rupturing the overstrained capillaries, and the consequent outpouring of the various constituents of the blood.

Quite perceptible elongation of these teeth occur, becoming more apparent as the inflammatory process continues.

Also more or less swelling of the soft parts adjacent appears as a constant symptom. Excessive pain is experienced upon the application of pressure or percussion, and sometimes even the slightest touch is intolerable. There being a continuous deep, dull and throbbing pain.

Structural changes are taking place and the quality of the blood is altered; suppuration is in progress.

What is termed the sac, and by some the pus secreting sac, is formed from the vitiated blood, and is no more nor less than a mass of coagulated lymph.

The alveolus is absorbed from the undue pressure, and the structural changes which are occurring; formation of pus increases until an outlet is effected for its escape.

Quite a variation exists in the character of the discharge from alveolar abscess in different cases, and at different stages of the same case. Sometimes consisting of pure or laudable pus, but generally it presents less the character of pus and is a vitiated, ichorous fluid. It being modified by the systemic condition, by the tissue disintegrated, and by the character of the local irritants.

The size of the abscess cavity varies, owing to the severity of the disease, and the peculiar susceptibility of the parts.

Sometimes the cavity enlarges after the pus has escaped, though generally it reaches its full size before that time.

The pus being left to force for itself an avenue of escape, the discharge occurs in various directions, and at variable distances from the point of secretion.

At times it occurs through the root, or between the tooth and alveolus, at other times directly through the alveolus and gum.

Occasionally the abscess is formed within the antrum, when the root of the tooth nearly or quite perforates the floor of that cavity, and becomes complicated by involving its lining membrane. When this is the case the disease is difficult to treat, and frequently necessitates extraction of the offending member. Oftentimes the fistulous opening is at some considerable distance from the seat of the difficulty, though always following some natural avenue, which furnishes the least resistance to the onward progress of the pent-up matter.

Alveolar abscess is exceedingly variable in character, owing to the constitutional idiosyncrasy of the patient, the condition of the surrounding parts and the exciting cause.

Patients of a manifest inflammatory diathesis are much more subject to abscess, or those in whom there is considerable local inflammation from some local exciting cause.

Sometimes after the formation of an abscess, when there is a discharge of healthy pus, nature, unaided, may effect a cure, but in constitutions of a cachectic diathesis the discharge is very apt to continue and is of a purulent, acrid character.

Often quite serious consequences follow neglect of these cases, as necrosis of the roots of teeth and portions of maxilla, and disease of the antrum; bad breath, dyspepsia, neuralgia and the like, being frequent concomitants of the disease.

*Treatment.* Obtaining a minute history of the case and making a thorough inspection, not only of the affected organ and its surroundings, but of the general appearance of the patient, and determining his constitutional peculiarities, will aid greatly in making a proper diagnosis and instituting a systematic course of treatment.

In cases of threatened abscess the indications are for removal of the cause of irritation and general antiphlogistic treatment.

When the abscess is of recent origin a cure can much more readily be effected, than in one of long standing.

The first step in chronic, as in acute cases, will be to evacuate the pus cavity and the root canal of their contents, enlarging the latter to the apex of the root, and thoroughly cleanse these applying disinfectants.

The object of the local treatment being to break up and destroy the accumulated lymph mass.

This is accomplished either by therapeutic or surgical means, or by the two combined.

If therapeutic treatment alone is employed, the agent to be used is introduced through the apex of the root to the seat of the disease. In order to break up an abscess by an operation, it must be easy of access, and scarcely ever can it be performed through the root.

But in the great majority of cases where the discharge is through the root, therapeutic treatment alone will suffice.

When an opening can not be made through the gum opposite the point of the root, in accessible cases, either by stopping up the root and causing the pus to force an opening, or by at once making an incision with a sharp pointed bistoury, and thus reach the seat of the disease. When there is already an opening through the alveolus and gum, the canal should be sufficiently enlarged to admit of free use of the instrument in dissecting the lymph mass from the point of the root, removing it as completely as possible.

If the case is a favorable one, nature alone will restore the part to health. But generally therapeutic treatment will be required to assist in the work.

The agent to be used should be forced up through the apex of the root, and, if possible, out at the opening in the gum. A pledget of cotton should be kept in the fistulous opening to allow of free drainage and enable it heal from the bottom. These applications should be repeated every day or two until the discharge ceases.

Abscess of the inferior maxilla is much more difficult to treat, from a want of free egress for the secretion, and in many of these cases removal of the tooth seems to be indicated.

And even in many cases of the upper maxilla, the most prompt and efficient treatment consists in the operation of replantation.

In performing this, the tooth should be carefully extracted to avoid fracture of the alveolus or laceration of the gums; then remove from the root or roots all the diseased periosteum and coagulated lymph; also the end of the root should be excised and rounded.

The cavity of decay, the pulp chamber and root canal should be thoroughly cleansed, formed and permanently filled. Upon the removal of the tooth a pledget of cotton should be placed in the socket, either moistened with some preparation to prevent, to a certain extent, coagulation of the blood, while working at the tooth, or allowing the blood to coagulate in the socket and remove just before replacing the tooth.

It now being ready for insertion, the cotton is to be removed and the socket cleansed of the clots of blood, and all that does not properly belong there.

Then carefully replace the tooth, allowing the jaws to be closed which will carry it to its precise position, and apply stays or ligatures if necessary. Usuallv the tooth will become firmly attached in a few days.—*Transactions Michigan Dental Association.*

